# Emergence of *bla*_NDM-7_–Producing *Enterobacteriaceae* in Gabon, 2016 

**DOI:** 10.3201/eid2302.161182

**Published:** 2017-02

**Authors:** Mesmin Moussounda, Seydina M. Diene, Sandra Dos Santos, Alain Goudeau, Patrice François, Nathalie van der Mee-Marquet

**Affiliations:** Hôpital d’Instruction des Armées Omar Bongo Ondimba, Libreville, Gabon (M. Moussounda);; Centre Hospitalier Régional Universitaire, Tours, France (M. Moussounda, S. Dos Santos, A. Goudeau, N. van der Mee-Marquet);; University of Geneva Hospitals, Geneva, Switzerland (S.M. Diene, P. François)

**Keywords:** *bla*_NDM-7_, *Klebsiella pneumoniae*, *Enterobacter cloacae*, carbapenem-resistant *Enterobacteriaceae*, carbapenemase-producing *Enterobacteriaceae*, Gabon, Central Africa, Africa, bacteria

## Abstract

Reports of carbapenemase-producing *Enterobacteriaceae* in Africa remain rare and assess mostly *bla*_OXA-48_–producing isolates from Mediterranean countries and South Africa. We identified *bla*_NDM-7_–producing *Enterobacteriaceae* in Gabon in 2016. The isolates contained *bla*_NDM-7_ IncX3 plasmids that were unusual and similar to the one described in a colistin-resistant *Klebsiella pneumoniae* SZ04 isolate from China.

Carbapenems are used as last-line antimicrobial drugs for treating infections caused by multidrug-resistant gram-negative bacilli. Their effectiveness is challenged by the emergence of carbapenemase-producing *Enterobacteriaceae* (CPE). A new type of β-lactamase, *bla*_NDM_, was reported from a patient in Sweden in 2007 (*1*). Since then, *bla*_NDM_ CPE have been identified worldwide and described as endemic to the Indian subcontinent and the Balkans (*2*). In countries to which they are nonendemic, CPE are reported mainly from patients with a history of hospitalization in a CPE-endemic area and, more rarely, in patients without history of travel (*3*). Reports on CPE in Africa are scarce, likely because monitoring of antimicrobial resistance remains uncommon. *bla*_OXA-48_ and *bla*_NDM-1_ CPE have been reported from the Maghreb area, Nigeria, Kenya, and South Africa, and single cases of *bla*_NDM-4_ and *bla*_NDM-5_ *Escherichia coli* have been reported in Cameroon, Algeria, and Uganda (*2*,*4*,*5*).

In January 2016, we conducted a point-prevalence study in all patients at the military general hospital of Libreville, Gabon. We collected demographic and clinical data and screened patients by rectal swabbing with Amies medium transport swabs (Copan Italia SPA, Brescia, Italy). The swabs were placed in 0.5 mL sterile water; 0.1 mL of the resulting suspension was streaked onto a selective agar plate provided for the identification of bacteria resistant to third-generation cephalosporins (CHROMagar, Paris, France). The plates were incubated for 48 h at 35°C. We used matrix-assisted laser desorption/ionization time-of-flight mass spectrometry technology (Brucker, Bremen, Germany) to confirm each isolate thought to be *Enterobacteriaceae*. 

We performed antimicrobial drug susceptibility testing by the agar disk diffusion method (http://www.eucast.org/). Isolates resistant to second- and third-generation cephalosporins were investigated for MIC of ertapenem using Etest (bioMérieux, Marcy-L’Étoile, France) and for carbapenemase production by the CarbaNP test (bioMérieux). For molecular characterization, we performed Sanger sequencing of PCR amplicons of the gene. Purified genomic DNA of the *bla*_NDM_-producing isolates was subjected to whole-genome sequencing on a HiSeq system (Illumina, San Diego, CA, USA). Reads were filtered for quality with fastq-mcf (Ea-utils: http://code.google.com/p/ea-utils). We used Edena version 2 for genome assembly (*3*); the genome was annotated by the National Center for Biotechnology Information pipeline. The resistome of the isolates was investigated through the ARG-ANNOT database. 

The study was performed in accordance with French and Gabonese recommendations. Ethical approval was obtained at the local level. 

We enrolled a total of 138 patients (84 women, 54 men; median age 32 years) in the study. The population had poor health status (a fatal illness likely to occur within the next 5 years for 35.5%; cancer and immunodepression in 5.8% and 22.5%, respectively). Recent hospitalization and antimicrobial drug therapy were reported for 32.5% and 15.9% of patients, respectively. CPE carriage was found in 7 (5.1%) patients and was associated with neonates (p<0.001). The CPE isolates harbored multiple antimicrobial resistance genes (but no *mcr-1* or *mcr-*2) and remained susceptible to tigecycline. 

Three isolates harbored *bla*_OXA-48_ and 4 a *bla*_NDM-7_ determinant (LC154935.1) (Table). *bla*_NDM-7_ is an infrequent allele described in Singapore and in patients returning from India (*2*,*6*) and was recently identified in patients not connected to a CPE-endemic area (*3*) and in nosocomial outbreaks (*7*,*8*). *bla*_NDM_ determinants linked to *ble*_MBL_ are frequently described on conjugative self-transferable IncX3 plasmids. Genome sequence analysis of the 4 Gabon isolates revealed *bla*_NDM-7_ and *ble*_MBL_ carried within a transposon element on a plasmid differing from the *bla*_NDM_ IncX3 plasmids carried on India isolates but highly similar (20 single-nucleotide polymorphisms) to an IncX3 plasmid in a *mcr*-1 *bla*_NDM-5_–producing *Klebsiella pneumoniae* strain isolated from a patient from China (*9*). The 4 patients from whom these isolates were obtained (3 neonates and an 83-year-old woman from the orthopedic unit) had no epidemiologic link with any foreign countries; their acquisition of the *bla*_NDM-7_ CPE was unexplained.

**Table:** Characteristics of *bla*_NDM-7_–producing *Enterobacteriacae* isolates from patients in a military hospital, Gabon, 2016*

Characteristic	Isolate			
	S1	S2	S3	S4
Species	*Klebsiella pneumoniae*	*K. pneumoniae*	*K. pneumoniae*	*Enterbacter cloacae*
No. contigs	83	104	86	61
Genome and plasmid size, bp	5′688′′217	5′710′′179	5′699′′938	4′860′′761
Resistome genes according to antibiotic class				
β-lactams				
*bla*_SHV-28_	+	+	+	
*bla*_CTX-M-15_	+	+	+	
*bla*_NDM-7_	+	+	+	+
*bla*_OXA-9_	+	+	+	
*act*-17				+
*amp*R				+
*lap*-2				+
*bla*_SHV-12_				+
*bla*_TEM-104_				+
Aminoglycosides				
*aac*6′-Ib	+	+	+	
*aac*3-IIa	+	+	+	
*ant*3	+	+	+	
*aac*3				+
Sulfamids				
*fol*A	+	+	+	+
*sul*1	+	+	+	
*sul*2	+	+	+	+
*dhfr*1	+	+	+	+
Others				
*fos*A	+	+	+	
* arr-ms*	+	+	+	
*tet*D	+	+	+	
*tet*A-2				+
*qnr*S1				+
* mphA*				+
* cat*				+

*aac3, aminoglycoside 3-N-acetyltransferase; *aac*3-IIa, aminoglycoside N-acetyltransferase AAC(3)-IIa; *aac*6′-lb-cr, AAC(6′)-Ib-cr family aminoglycoside N(6′)-acetyltransferase; *aac*6′-Ib, AAC(6′)-Ib family aminoglycoside 6′-N-acetyltransferase; *act*-7, cephalosporin-hydrolyzing class C β-lactamase ACT-17; *amp*R, ampicillin chromosomal-mediated β-lactamase; *ant*3, streptomycin 3′′-adenylyltransferase; *arr-ms*, rifampin ADP-ribosyl transferase; *bla*_CTX-M-15_, class A extended-spectrum β-lactamase CTX-M-15; *bla*_NDM-7_, New Delhi metallo-β-lactamase NDM-7; *bla*_OXA-9_, oxacillin-hydrolyzing class D β-lactamase OXA-9; *bla*_SHV-12_, β-lactamase SHV-12 ; *bla*_SHV-28_, class A β-lactamase SHV-28; *bla*_TEM-104_, β-lactamase TEM-104; *cat*, chloramphenicol acetyltransferase; *dhfr*1, dihydrofolate reductase type 1; *fol*A, dihydrofolate reductase; *fos*A, fosfomycin resistance glutathione transferase FosA; *lap*-2, class A β-lactamase LAP-2; *qnr*S1, quinolone resistance pentapeptide repeat protein QnrS1; *mph*A, Mph(A) family macrolide 2′-phosphotransferase; *sul*1, sulfonamide-resistant dihydropteroate synthase Sul1; *sul*2, sulfonamide-resistant dihydropteroate synthase Sul2; *tet*A-2, tetracycline resistance protein, class C; *tet*D, tetracycline efflux MFS transporter Tet(D).

Because of the social and economic relationships between China and Gabon, the travels of asymptomatic CPE carriers from China to Gabon can be expected to have facilitated the spread of CPE in Gabon. Several multidrug-resistant clones of *K. pneumoniae*, including sequence type 307 (*10*), have been recognized as having emerging epidemic potential worldwide. The genome analysis of the 3 *bla*_NDM-7_–producing *K. pneumoniae* isolates from Gabon revealed clonal isolates (2 and 5 single-nucleotide polymorphisms between them) of sequence type 307. This result suggests an uncontrolled spread in the hospital intensive care unit.

This description of *bla*_NDM-7_ in Africa highlights the international dissemination of carbapenemase determinants and the combination of 2 aggravating factors, resulting in an alarming situation: the identification of *bla*_NDM-7_ within a transposon element on a conjugative plasmid with a potentially very high level of transmissibility, and the implication of the presence of *K. pneumoniae*, a pathogen with a high potential to persist and disperse in the hospital environment. Urgent measures are required, including the rational use of antimicrobial drugs, public education on the importance of hygiene, and diligent surveillance to control the spread of these multidrug-resistant organisms in the hospital setting.
